# Surgical Approach for TAVI Replacement in Endocarditis: A Descriptive Technique

**DOI:** 10.3390/jcm14072348

**Published:** 2025-03-29

**Authors:** Sébastien D’ulisse, Karim Homsy, Sotirios Marinakis, Serge Cappeliez, Badih El Nakadi

**Affiliations:** 1 Marie Curie Hospital, Université Libre de Bruxelles (ULB), 6042 Charleroi, Belgium; 2 Cardiac Surgery, Marie Curie Hospital, 6042 Charleroi, Belgium; karim.homsy@humani.be (K.H.); sotirios.marinakis@humani.be (S.M.); serge.cappeliez@humani.be (S.C.); badith.elnakadi@humani.be (B.E.N.)

**Keywords:** TAVI (transcatheter aortic valve implantation), endocarditis, surgical technique, prosthetic valve replacement, self-expanding valve

## Abstract

**Background/Objectives:** Transcatheter Aortic Valve Implantation (TAVI) has significantly improved the management of aortic valve disease, but post-TAVI infective endocarditis, occurring in 0.5–3.1% of cases, remains a serious complication. Due to a high mortality rate and technical challenges, surgical replacement of infected TAVI prosthetic valves is performed in only 11.4% of cases. **Methods:** This case describes a standardized surgical technique for the removal and replacement of self-expanding TAVI prosthetic valves in the case of infective endocarditis. **Results:** The proposed approach aims to facilitate valve explantation while minimizing surgical risks. **Conclusions:** We believe that this technique could be particularly beneficial for surgeons managing these complex cases, by reducing surgical complications and improving patient outcomes. Further studies are necessary to validate its long-term efficacy and applicability in broader clinical settings.

## 1. Introduction

Transcatheter aortic valve implantation (TAVI) was introduced in the early 2000s as an alternative to surgical aortic valve replacement [1]. It has revolutionized the management of aortic valve diseases, particularly in high-risk patients [2]. Despite its widespread adoption and favorable outcomes, infective endocarditis (IE) post-TAVI remains a rare but serious complication, occurring in 0.5% to 3.1% of cases [3,4], a rate comparable to that observed in conventional surgical valve replacement [5,6].

Endocarditis after cardiac surgery presents unique challenges, requiring a nuanced grasp of its temporal onset and causative microbial agents. Early-onset cases emerge shortly after surgery, constituting approximately 30% to 40% of all cases [7]. They are often associated with nosocomial pathogens such as Staphylococcus aureus and Enterococci. In contrast, late-onset endocarditis occurs months to years after implantation and is more frequently caused by less virulent organisms such as Viridans group streptococci and coagulase-negative staphylococci [8].

In post-TAVI infective endocarditis, Enterococci are the most commonly isolated pathogens, accounting for 25.9% of cases, followed by Staphylococcus aureus in 16.1% [9]. Although rare, post-TAVI infective endocarditis is associated with high mortality, reaching up to 39% [10]. It presents significant diagnostic and therapeutic challenges. Surgical replacement of infected TAVI prosthetic valves requires a meticulous approach due to the complexities of device removal, infection control, and subsequent valve replacement.

Post-TAVI infective endocarditis presents with varied clinical symptoms, ranging from nonspecific to acute manifestations such as fever, heart failure, or embolic stroke, particularly in elderly patients. Heart failure occurs in over 50% of cases, while approximately 20% of patients exhibit nonspecific symptoms, like malaise, weakness, or weight loss [5]. Unlike native valve endocarditis, high-grade fever and heart murmurs are less prevalent in post-TAVI cases. Echocardiographic diagnosis is particularly challenging due to the valve’s metallic components, which can cause reflectance and shadowing artifacts, limiting the detection of small vegetations [11].

With the increasing number of TAVI procedures performed worldwide, the incidence of this complication is expected to rise [12]. Although guidelines recommend early surgery for complicated cases, it is rarely performed.

This case report aims to provide a detailed description of our surgical approach for the replacement of a self-expanding TAVI prosthetic valve in the setting of infective endocarditis.

## 2. Materials and Methods

### Case Report:

**Figure 1:**
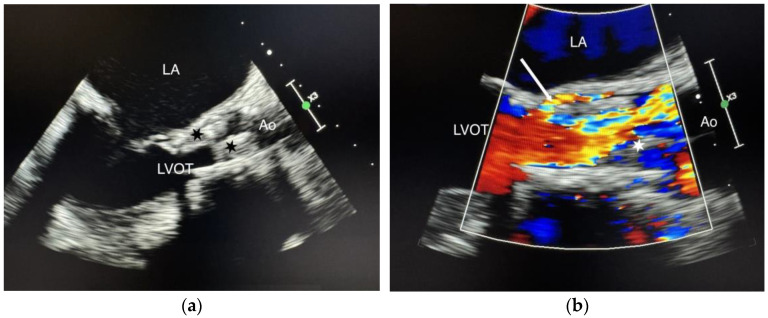
(**a**) Standard TEE showing the vegetations. Cusp enlargement and vegetations (black stars); (**b**) Color Doppler TEE demonstrating paravalvular regurgitation. Vegetation (white star), paravalvular regurgitation (white arrow), LA (left atrium), LVOT (left ventricular outflow tract), Ao (aorta).

We present the case of a 75-year-old female patient, with multiple cardiovascular comorbidities, admitted to the hospital due to deterioration of her general condition. She presented with fever, asthenia, anorexia, and loss of mobility. Ten months prior to her admission, she underwent a TAVI with a self-expanding prosthetic valve (Navitor, 27 mm, Abbott Cardiovascular, Abbott Park, IL, USA). Blood cultures were positive for a multi-sensitive Enterococcus faecalis. Transesophageal echocardiography (TEE) demonstrated a 2 cm mobile mass on the aortic prosthesis leaflets, with a mean gradient of 49 mmHg and a normal left ventricle ejection fraction. Intravenous antibiotic therapy with amoxicillin-clavulanate plus ceftriaxone was initiated. One month later, TEE showed no change in the vegetation size or the mean transvalvular gradient (Figure 1a,b).

White blood cell count increased to 14,400/mm^3^ and CRP levels reached 108 mg/L. After evaluation by a multidisciplinary team including a cardiologist, an interventional cardiologist, an infectious disease specialist, and a cardiac surgeon, surgical valve replacement was decided.

## 3. Results

### Technical Description:

**Figure 2:**
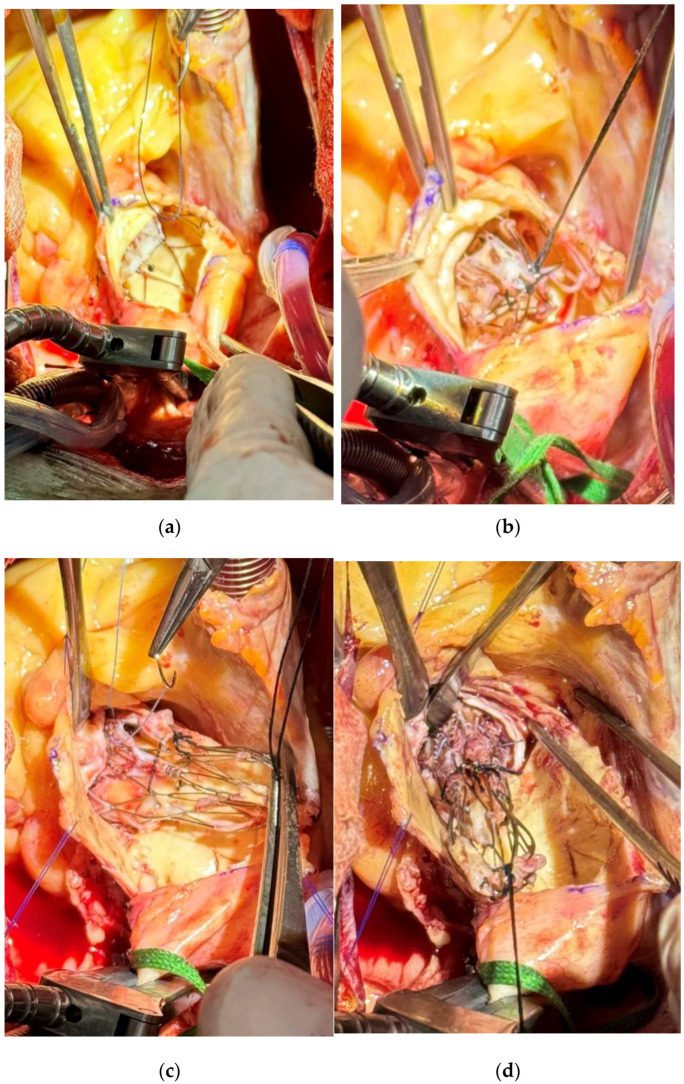
(**a**) Initial exposure and first stitch on the last cell row of the implanted prosthesis after dissection of the aortic wall. (**b**) Crimping of the first silk suture on the upper part of the implanted prosthesis. (**c**) Placement of the third suture on the lower part of the implanted prosthesis. (**d**) Extraction of the infected prothesis valve with a careful detachment to minimize trauma to the aortic annulus.

After aortic cross-clamping, a hockey-stick incision was initiated just above the Navitor prosthesis (Figure 2a). The last cell row of the stent was gently dissected from the aortic wall and a silk stitch was passed through those cells and tied in order to crimp the distal end of it (Figure 2b).

The dissection was gradually prolonged proximally as well as the aortotomy. A second crimping stitch was passed through the second cell row (Figure 2c), allowing an easy mobilization of the stent and then easier proximal dissection. After a third similar stitch was added on the proximal cell row of the stent and the Navitor valve completely crimped, it was delicately detached from the aortic annulus without any damage (Figure 2d).

**Figure 3:**
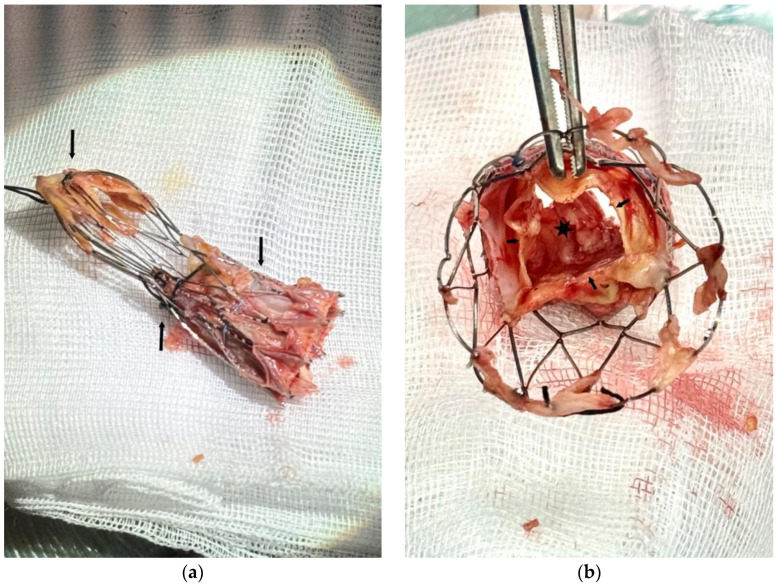
(**a**) Self-expanding valve after successful removal with the three crimping stitches (arrows). (**b**) View from the aortic side of the valve. Leaflets free margin (small arrows). Obstructing vegetation on the ventricular aspect of the cusps (black star).

In Figure 3a, we can see the self-expanding valve after successful removal. A large vegetation obstructing the LVOT was found on the ventricular side of the cusps Figure 3b.

The bulky native cusps were removed and an annular enlargement using the Manouguian technique was used. An Edwards Inspiris, 21 mm, pericardial valve was implanted, and the patient was easily weaned from bypass.

The patient remained in the intensive care unit for 3 days and was discharged to a rehabilitation unit 10 days after surgery. On long-term follow-up, transthoracic echocardiography (TTE) showed no abnormalities.

## 4. Discussion

Our case highlights the surgical approach for the treatment of infective endocarditis after TAVI, a rare but serious complication.

Recent studies by Khan A. et al. [9] have emphasized the high mortality and complications associated with post-TAVI infective endocarditis. It is noteworthy that the majority of post-TAVI endocarditis cases are currently treated with antibiotic therapy. Surgical interventions remain rare, accounting for approximately 11.4% of cases [5].

Both TTE and TEE have reduced sensitivity and specificity in detecting prosthetic valve endocarditis (PVE) [13], which is particularly true for IE related to TAVI. A major challenge is the early and accurate diagnosis, particularly in patients with negative TTE and TEE but with persistent bacteremia after a TAVI (Mangner et al. [13]).

Recent data suggest that multimodal imaging techniques, such as 18F-FDG PET/CT, could improve the diagnostic sensitivity for IE, especially in cases where conventional echocardiographic results are inconclusive [14]. Incorporating these imaging modalities into the diagnostic algorithm could allow for earlier identification of patients requiring surgical intervention.

Surgical approaches remain poorly defined given the limited number of cases described in the literature. Our technique offers a simple and reproducible method for a step-by-step explantation of self-expanding TAVI valves.

Although surgery remains the cornerstone of treatment for carefully selected patients, promoting a multidisciplinary collaboration is crucial. Collaboration between cardiac surgeons, interventional cardiologists, infectious disease specialists, and radiologists could improve diagnosis, management, and prognosis in this at-risk population.

## 5. Conclusions

Post-TAVI endocarditis remains a complex condition with significant diagnostic and therapeutic challenges, particularly in high-risk patients. Our case demonstrates the successful surgical management of an infected self-expanding TAVI valve using a standardized approach, which could provide useful guidance for surgeons dealing with similar rare cases. However, this technique requires further validation. Future studies on a larger cohort are essential to assess its broader applicability and establish standardized surgical protocols.

## Data Availability

The original contributions presented in this study are included in the article. Further inquiries can be directed to the corresponding author(s).

## Abbreviations

The following abbreviations are used in this manuscript:

TAVI	Transcatheter aortic valve implantation
IE	Infective endocarditis
TEE	Transesophageal echocardiography
LVOT	Left ventricular outflow tract
TTE	Transthoracic echocardiography
PVE	Prosthetic valve endocarditis
LA	Left atrium
AO	Aorta
